# Case report: Pyrotinib and tegafur combined with radiotherapy achieved notable response in HER2-amplified rectal cancer with multiple metastases after multiline treatments

**DOI:** 10.3389/fphar.2024.1431542

**Published:** 2024-08-13

**Authors:** Peng Huang, Feng Wen, Xin Wang

**Affiliations:** ^1^ Division of Abdominal Tumor Multimodality Treatment, Cancer Center, West China Hospital, Sichuan University, Chengdu, Sichuan, China; ^2^ Department of Medical Oncology, Cancer Center, West China Hospital, Sichuan University, Chengdu, Sichuan, China; ^3^ Department of Radiation Oncology, Cancer Center, West China Hospital, Sichuan University, Chengdu, Sichuan, China

**Keywords:** rectal cancer, HER2, pyrotinib, multiline treatments, radiotherapy

## Abstract

Metastatic colorectal cancer (mCRC) is characterized by significant phenotypic heterogeneity at the molecular level and presents a poor prognosis. Chemotherapy is commonly employed as the primary treatment option. Nevertheless, the advantages of chemotherapy are constrained, underscoring the critical necessity for novel treatment protocols aimed at enhancing patient outcomes. Human epidermal growth factor receptor 2 (HER2) has been recognized as a promising therapeutic target in mCRC. Pyrotinib, an innovative irreversible dual tyrosine kinase inhibitor targeting HER2, effectively inhibits cancer progression in various types of human cancers. Here, we present a case of a 39-year-old female with metastatic rectal cancer showing amplification of HER2. Despite resistance to multiple therapies, including trastuzumab and pertuzumab, the patient exhibited a remarkable therapeutic response to pyrotinib, tegafur combined with radiotherapy. This case provides evidence for the feasibility and potential efficacy of deploying pyrotinib in the salvage treatment of mCRC patients with HER2 amplification even though resistant to other anti-HER2 drugs.

## 1 Introduction

Colorectal cancer (CRC) represents a significant contributor to cancer-related morbidity and mortality worldwide, with rectal cancer cases estimated to be 46,220 in the United States (Shin et al., 2023; Siegel et al., 2024). Approximately 25%–40% of rectal cancer patients are represented with distant metastases at initial diagnosis, which is the primary cause of therapy failure (van Gijn et al., 2011; Fokas et al., 2014). Rectal cancer holds a high potential for metastasizing to the liver, lung, and bone (He et al., 2023). Brain metastases represent the most prevalent type of intracranial tumors in adults, yet they infrequently originate from rectal cancer (Boire et al., 2020).

Multidrug combination regimens such as FOLFOX and FOLFIRI have been recognized as standard therapies for metastatic rectal cancer (Biller and Schrag, 2021). Nevertheless, chemotherapy has significant ceiling effects in the context of long-term toxic events and multi-drug resistance (Kim, 2015). In recent years, advances in comprehending the molecular and genetic complexities of cancer have paved the way for introducing more effective treatment modalities into the later-line treatments for rectal cancer, out of which molecularly targeted therapy has been well-characterized and plays a crucial role in clinical management (Ohishi et al., 2023).

**FIGURE 1:**
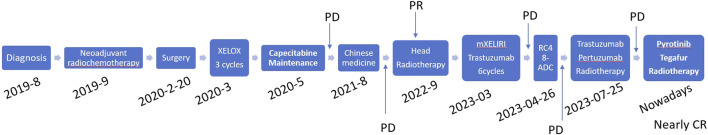
Time diagram of the treatment from August 2019 to May 2024 of the patient. PD, progressive disease; CR, complete response.

Human epidermal growth factor receptor 2 (HER2), encoded by the oncogene *ERBB2*, belongs to the epidermal growth factor receptor (EGFR) family (Oh and Bang, 2020). Many other solid tumors exhibit high levels of HER2 expression, including gastric cancer, urothelial carcinoma, lung cancer and biliary tract cancer (BTC), and anti-HER2 drugs have exhibited great therapeutic benefits in clinical settings (Lamberti et al., 2020; Mollica et al., 2020; Ricci et al., 2021; Rizzo et al., 2021). For example, in the global basket study SUMMIT, for nine patients with ERBB2-mutated BTC, the overall response rate (ORR) was 22% (Hyman et al., 2018). Additionally, a partial response (PR) was observed in a patient with HER2-amplified gallbladder cancer in a phase I study with the HER2-targeted bispecific antibody ZW2567 (Oh and Bang, 2020). Evidence has revealed that HER2 amplification comprises approximately 2%–11% of metastatic CRC (mCRC) patients with poor prognosis (Greally et al., 2018). The potent antitumor activity of HER2-targeted inhibitors on patients with HER2 amplification has been presented (Greally et al., 2018). Regarding evidence from clinical guidelines, trastuzumab plus pertuzumab has been recommended as the late-line treatment for HER2-amplified metastatic CRC that becomes refractory to chemotherapy (Meric-Bernstam et al., 2019). Pyrotinib is an orally administered, irreversible tyrosine kinase inhibitor (TKI) of the pan-ErbB receptor, which has been authorized for the therapy of HER2-amplified breast cancer in recurrent and neoadjuvant settings (Blair, 2018). Moreover, it exerts a potent antitumor effect in other HER2-amplified solid tumors including lung cancer and gastric cancer (Li et al., 2017; Blair, 2018; Niu et al., 2023). Herein, we report a rare case of rectal cancer harboring HER2 amplification with multiple metastases in the brain, scalp, lung, pancreatic head, and both kidneys, who has been treated with standard chemotherapy and several HER2 inhibitors, finally achieved notable response (nearly complete response) from pyrotinib and tegafur combined with radiotherapy (Figure 1).

## 2 Case presentation

In August 2019, a 39-year-old female patient was admitted to our hospital presenting with a constellation of intestinal symptoms including changes in stool consistency, bloody stool, and tenesmus. The patient was diagnosed with pulmonary tuberculosis 10 years ago. After undergoing anti-tuberculosis treatment, the condition improved. In addition, the patient has no history of smoking or alcohol consumption and has no family history of malignant or genetic diseases. After being seen by the attending physician, the patient underwent further relevant examinations. Whole-abdominal enhanced CT has revealed a lobulated soft tissue mass of 3.0 cm × 2.2 cm in the rectal wall. Chest CT showed bilateral old pulmonary tuberculosis. Then, the patient underwent a proctoscopy and biopsy. At a distance of 3–6 cm from the anal verge, an ulcerative-type neoplasm is observed, with a congested and ulcerated base, surrounded by mucosal ramparts resembling a dam, involving approximately half of the circumference of the lumen, leading to relative luminal narrowing. The histopathologic examination revealed high-grade intraepithelial neoplasia and mucosal intraepithelial carcinoma formation. The tumor stage was cT3N0M0. Then the patient accepted a neoadjuvant chemo-radiotherapy regimen comprising four treatment cycles of mFOLFOX6 (oxaliplatin, leucovorin, and fluorouracil), one treatment cycle of XELOX, (oxaliplatin and capecitabine) along with external irradiation with a dosage of 50.4Gy/28f. After that, an imaging examination revealed the significant shrinkage of the tumor.

On 20 February 2020, the patient underwent low anterior resection for rectal cancer. Postoperative histological examination confirmed the diagnosis of moderately differentiated adenocarcinoma of the rectum, pT3N0M0, TRG2. Immunohistochemical analyses suggested “*MLH1* (+), *MSH2* (+), *MSH6* (+), *PMS2* (+), Ki–67:50%.” After surgery, three cycles of the mFOLFOX6 regimen were given in the adjuvant chemotherapy setting. Then, capecitabine maintenance therapy was followed in December 2020. Subsequent regular follow-ups showed no progression of the disease.

**FIGURE 2:**
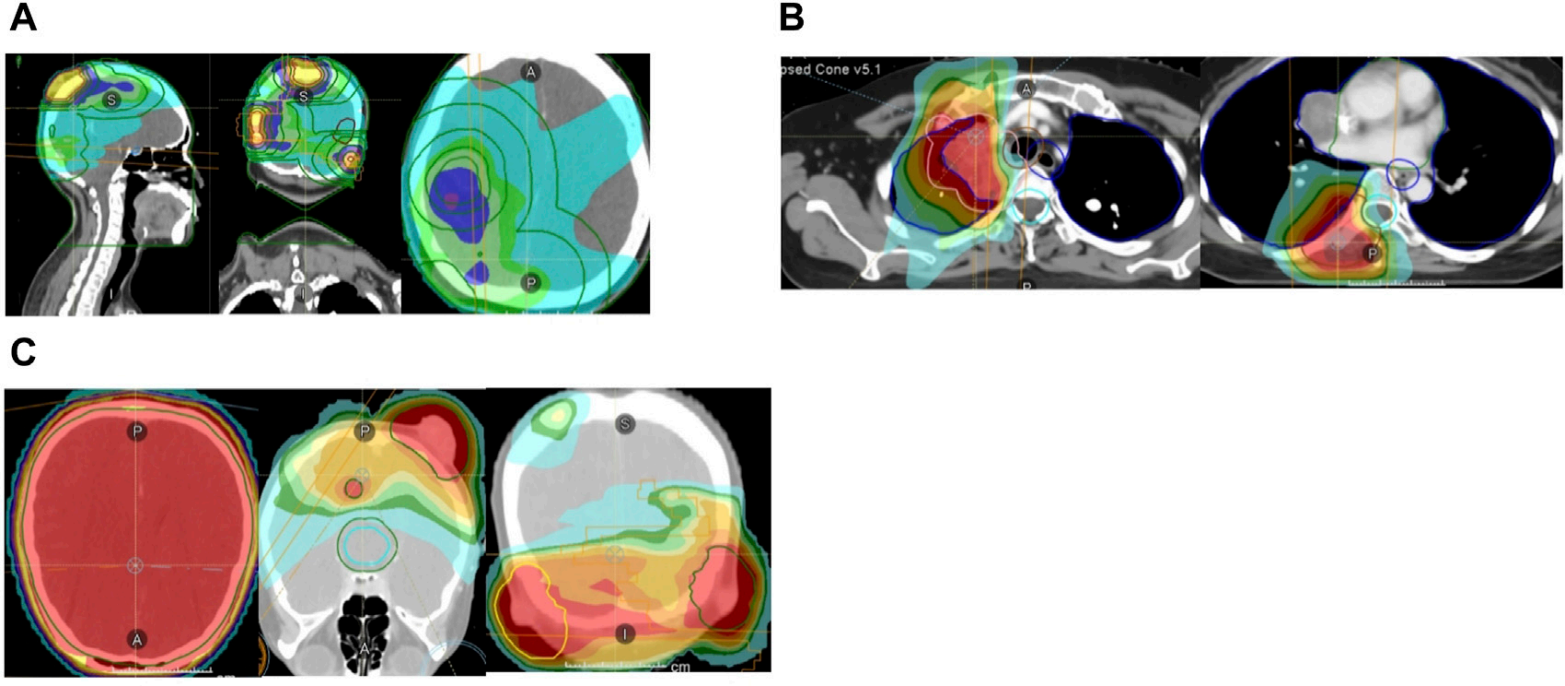
**(A)** Brain and scalp radiotherapy plans (51Gy/17f). **(B)** Mediastinum and vertebral body radiotherapy plans (mediastinum: 56Gy/8f, vertebral body: 40Gy/5f). **(C)** Whole brain radiotherapy (24Gy/8f) and additional dose of all intracranial and scalp lesions (24Gy/8f).

**FIGURE 3:**
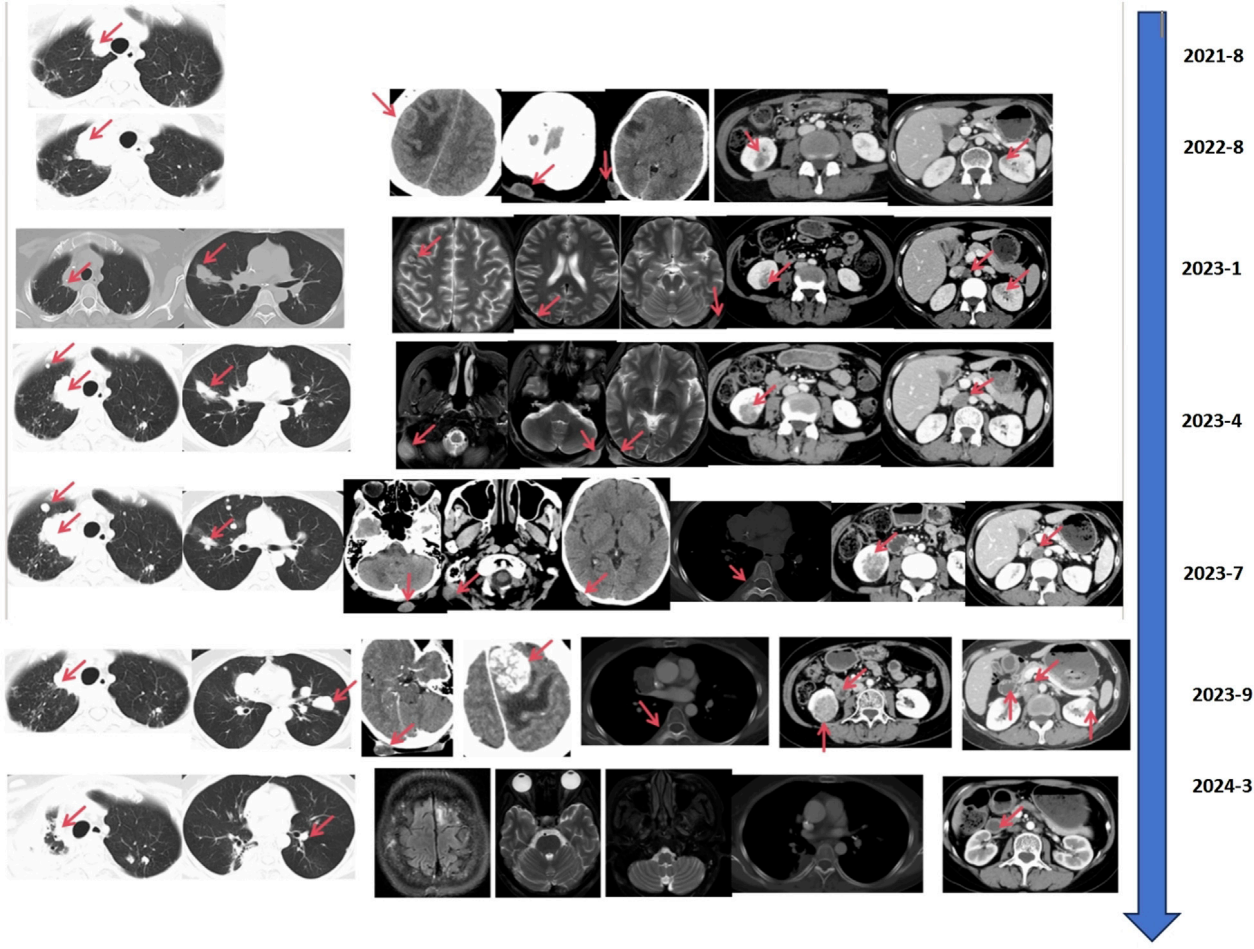
Clinical images of the patient.

In August 2021, the countercheck of chest CT showed several bilateral pulmonary nodules, while the biggest one was in the upper lobe of the right lung near the mediastinal soft tissue, which was about 1.8 cm × 1.5 cm in size, suggesting tumor metastasis. Palliative chemotherapy with mFOLFOX6 regimen was given for one cycle and the patient spontaneously discontinued the treatment. After that, the patient received treatment with Chinese herbs and was lost to follow-up. From March to June 2022, the patient continued to grow three masses on the scalp which were mistaken for abscesses by herself, and did not receive proper treatment. Then, a constellation of symptoms including intermittent head pain, nausea, vomiting, fatigue, left upper limb and facial twitches were presented. In August 2022, the patient was admitted to our hospital again. Enhanced CT revealed multiple nodules in the parieto-occipital regions and the largest one is about 2.5 cm × 1.5 cm accompanied by significantly increased tumor markers (CA199 > 1,000 U/mL, CEA >83.7 U/mL). Moreover, a 2.0 cm × 1.6 cm metastatic node was found in the right frontal cortex. Besides that, metastatic signs were found in both kidneys and the head of the pancreas and the metastatic lesion in the right upper lobe of the lung has increased in size compared to previous CT images. Biopsy of scalp metastases showed that skin metastases tended to originate from rectal cancer. Further immunohistochemistry showed *CK20* (+), *CDX2* (+), *CK7* (−), *CK5/6* (−), *TIF-1* (−), Ki–67: 80% and *HER2* diffuse positive. Capecitabine following the head radiotherapy (51Gy/17f) was performed (Figure 2A). After radiotherapy, the symptoms of headache and limb twitching have significantly improved. The CT re-examination revealed the shrunk tumor mass both in the scalp and brain, which indicated a great therapeutic response to radiotherapy. Then, the next-generation sequencing (NGS) (1,021 genes; Chengdu Huachuang Qide Medical laboratory Co., LTD., Chengdu, China) based on scalp specimens revealed the *HER2* amplification (copy number: 66.8), while *RAS* and *BRAF* were negative. The detailed results are listed in Table 1. Based on the NGS results, the second-line regime: six treatment cycles of mXELIRI (Irinotecan and capecitabin) combined with trastuzumab was initiated on 28 October 2022. The patient tolerated this new regime well, with no apparent side effects. During treatment, the best therapeutic effect is PR. In April 2023, progressive disease was evaluated based on the imaging manifestations of enlarged nodules of the lung, pancreatic head, centrum of the 7th thoracic vertebrae, right kidney and scalp, while paraaortic lymph node metastasis was also presented. The patient volunteered for a Phase II clinical study and was treated with six cycles of RC48-ADC on 26 April 2023. The RC48-ADC represents a new antibody-drug conjugate (ADC) targeting HER2, which specifically transports the cytotoxic compound monomethyl auristatin E (MMAE) to HER2-positive tumor cells (Hong et al., 2023). Previous clinical trials have demonstrated its good tolerability and potential effectiveness in HER2-positive advanced bladder cancer (Sheng et al., 2021; Hong et al., 2023). The disease condition was stable during treatment. Reexaminations from July 2023 indicated the progression of lung and scalp lesions along with hoarseness and back pain. Then the patient was withdrawn from the clinical trial. In August 2023, the patient received trastuzumab + pertuzumab, followed by mediastinal and pyramidal radiotherapy with a dosage of 56Gy/8f, and 40Gy/5f respectively (Figure 2B). However, the enhanced CT examination revealed obvious enlargement of the scalp tumor in September 2023. Then tegafur, pyrotinib combined with head radiotherapy (48Gy/16f) was recommended by the oncologists in October 2023 (Figure 2C). Notably, follow-up examination revealed significant shrinkage of scalp and brain metastases. Besides, the multiple metastases in the lung, pancreatic head, both kidneys and paraaortic lymph nodes were also dramatically shrunk (Figure 3). Until the submission of the case report, the patient had survived for over 33 months since post-operative recurrence and now continues to receive the combination treatment of pyrotinib and tegafur and a notable response (nearly complete response in brain and scalp metastases) had been achieved. Currently, close follow-up is still underway.

**TABLE 1:** Gene alterations of the NGS results based on the metastatic scalp specimens.

Genes	Variations	Copy number/abundance
*KRAS*	Negative	—
*NRAS*	Negative	—
*BRAFV600E*	Negative	—
*NTRK2*	Negative	—
*NTRK3*	Negative	—
*MLH1*	Negative	—
*MSH2*	Negative	—
*MSH6*	Negative	—
*PMS2*	Negative	—
*RNF43*	NM_017763.4 C.953-1GsT amplification	42%
*ERBB2*	NM_004448.2 amplification	66.8
*FGFR1*	NM_023110.2 amplification	5.2
*MCL1*	NM 021960.4 amplification	4.0
*CDK6*	NM_001145306.1 amplification	3.0
EGFR	NM_005228.3 amplification	3.0
RAD50	NM_005732.3 deficiency	1.4
WRN	NM_000553.4 deficiency	1.4

KRAS, kirsten rat sarcoma viral oncogene homolog; NRAS, neuroblastoma rat sarcoma viral oncogene homolog; BRAF, v-raf murine sarcoma viral oncogene homolog B1; HER2, human epidermal growth factor receptor 2; NTRK1, neurotrophic tyrosine kinase, receptor, type 1; FGFR1, fibroblast growth factor receptor 1; MCL1, myeloid cell leukemia-1; CDK6, cyclin-dependent kinase 6; EGFR, epidermal growth factor receptor; NGS, next-generation sequencing.

## 3 Discussion

*HER2*, encoded by the *ERBB2* gene, will be overexpressed when the *ERBB2* gene is amplified (Oh and Bang, 2020). *HER2* serves as a significant oncogenic driver in mCRC (Chitkara et al., 2023). Regimens involving dual anti-HER2 drugs have shown significant antitumor activity in the relapsed setting (Chitkara et al., 2023). The regime of trastuzumab in conjunction with lapatinib achieved an objective response in 8 (30%) of 27 patients with *HER2* amplification and *KRAS* exon 2 wild-type tumors in the HERACLES-A trail (Sartore-Bianchi et al., 2016). Notably, the median OS and PFS of 46 weeks (95% CI 33–68 weeks) and 21 weeks (95% CI 16–32 weeks) (Sartore-Bianchi et al., 2016). Nevertheless, almost all cases that achieved an objective response in this study developed acquired resistance, mirroring the dilemma observed in the Mypathway study (Sartore-Bianchi et al., 2016; Meric-Bernstam et al., 2019). Mypathway was a multiple-basket phase II study that assessed the antitumor activity of the regime of trastuzumab in combination with pertuzumab in patients with solid tumors (Meric-Bernstam et al., 2019). This study included 69 CRC patients with *KRAS* wild-type and *HER2*-overexpressed tumors, out of which 22 (31.9%) achieved objective responses. However, in the setting of *KRAS-*mutant CRC, the ORR was only 8%, which indicated the limited antitumor activity of trastuzumab plus pertuzumab (Meric-Bernstam et al., 2019). Trastuzumab deruxtecan is an antibody–drug conjugate that has been approved in metastatic, HER2-overexpressed gastric and breast cancer in the United States (Jørgensen, 2023). The DESTINY-CRC01 study was a signal arm, phase II clinical trial that demonstrated the clinical benefits of trastuzumab deruxtecan in 78 patients with RAS wild-type and HER2-expressed mCRC (Siena et al., 2021). Patients with HER2 3+ status received the highest ORR of 57.5% (95% CI 40.9–73.0) (Siena et al., 2021). Interestingly, the ORRs of trastuzumab deruxtecan treatment in patients with or without prior anti-HER2 therapy were 43.8% and 45.9% respectively, which showed the rationality of applying trastuzumab deruxtecan in the later line treatment after prior anti-HER2 treatment (Siena et al., 2021). Moreover, the multicenter DESTINY-CRC02 trial further assessed the antitumor response of trastuzumab deruxtecan in HER2-amplified mCRC patients (NCT04744831) (Zheng-Lin et al., 2023).

Although the anti-HER2 regime exhibits great value in cancer therapy, however, the drug resistance impedes its clinical application. It is acknowledged that *EGFR* and *HER2* activate the *RAS/RAF/ERK* pathway to promote cell division, as well as the phosphatidylinositol 3-kinase (*PI3K*)*/PTEN/AKT* pathway to facilitate cell growth and survival (Rusnak et al., 2001). Dysregulation in the downstream pathway leads to resistance to targeted agents. In HER2-overexpressed solid tumors, *PTEN* was identified as a predictive biomarker of trastuzumab resistance (Tekesin et al., 2019; Yokoyama et al., 2021). Belli et al. showed that abnormal changes in *MEK* and *PIK3CA* can give rise to resistance to anti-HER2 agents in mouse models of HER2-amplified CRC (Belli et al., 2019). However, in our case, activating mutations of *PIK3CA* or *MEK* and decreased expression of *PTEN* was not detected in scalp metastasis samples. Notably, the amplification of fibroblast growth factor receptor 1 (*FGFR1*) was observed in the NGS testing. *FGFR* family of receptor tyrosine kinases mediate various cellular processes by dimerizing and activating the downstream signal network, including mitogen-activated protein kinase (*MAPK*) and *PI3K/AKT* (Sami and Karsy, 2013). María et al. revealed that *FGFR1* amplification was associated with a poor response to anti-HER2 treatment (Gaibar et al., 2022). In the cohort comprised breast cancer patients who received trastuzumab and pertuzumab, the pathological complete response rate was lower among those harboring FGFR1 amplification (Gaibar et al., 2022). Moreover, Ariella et al. reported that lapatinib + trastuzumab-resistant tumors exhibited increased *FGFR* phosphorylation, which led to significant stromal alterations in the tumor microenvironment and decreased tumor uptake of drugs (Hanker et al., 2017). These findings indicate that abnormal activation of the *FGFR* pathway is linked to primary or intrinsic resistance to therapeutic blockade of HER2.

Pyrotinib inhibits the autophosphorylation of the HER2, therefore suppressing the *MAPK* and *PI3K/Akt* signaling pathways (Blair, 2018). Several studies have demonstrated that the dual anti-HER2 regime of pyrotinib in combination with trastuzumab has shown significant anti-tumor activity in HER2 amplification, *RAS* wild-type mCRC patients who have become refractory to chemotherapy (Chang et al., 2022; Fu et al., 2023; Zhou et al., 2023). In our case, despite the resistance to trastuzumab + pertuzumab, pyrotinib demonstrated a remarkable clinical response. While trastuzumab and pertuzumab exclusively target HER2, pyrotinib also significantly inhibits *EGFR* signaling (Blair, 2018; Han et al., 2022). Furthermore, pyrotinib has shown high selectivity when assessed against a wide range of diverse kinases, including c-Kit, platelet-derived growth factor receptor β, kinase insert domain receptor, c-Src, and c-Met (Li et al., 2017). Thus, pyrotinib might manifest diverse effects through multiple mechanisms that differ from those of previous anti-HER2 medications, which could partially account for the exceptional response to pyrotinib in our case.

Interestingly, HER2 has been reported to be linked with adaptive radiation resistance, while HER2 inhibition is a promising strategy for overcoming radioresistance (Khan et al., 2024). Pyrotinib treatment reduced the cyclin D1 and cyclin-dependent kinase 4 (CDK4) levels for increasing G0/G1 arrest, resulting in the enhanced anti-proliferation effects of radiotherapy in esophageal cancer cells (Lian et al., 2020). Niu et al. discovered that pyrotinib in combination with radiotherapy achieved more tumor remission in the xenograft model (Niu et al., 2023). Mechanistic investigations have revealed that pyrotinib blocked the activation of the *ERK* signaling pathway mediated by radiation (Niu et al., 2023). Moreover, pyrotinib also promoted DNA damage, induced cell apoptosis, and enhanced senescence, which contributed to increased radiosensitivity (Niu et al., 2023). Brain metastasis frequently leads to mortality in patients with HER2-positive breast cancer. The standard treatment predominantly involves whole-brain radiotherapy (Kim et al., 2024). Tian et al. assessed the effectiveness of pyrotinib in conjunction with WBRT in patients with HER2-positive brain metastatic breast cancer (Tian et al., 2022). The oral administration of pyrotinib, combined with radiotherapy, significantly enhanced the ORR, and PFS in patients with HER2-positive brain metastases, without increasing adverse events (Tian et al., 2022). Furthermore, pyrotinib improved the radiosensitivity of HER2-positive breast cancer cell lines cultured *in vitro* (Tian et al., 2022). The findings suggest that pyrotinib could be an efficacious agent to increase tumor radiosensitivity and improve clinical prognosis in patients with HER2-positive brain metastatic breast cancer. Moreover, small TKIs such as pyrotinib has been demonstrated to possess enhanced blood-brain barrier permeability and specific anti-intracerebral tumor efficacy (Garcia-Alvarez et al., 2021). Besides, radiotherapy modifies the blood-brain barrier permeability, leading to an increase in the influx of drugs into the brain (Li et al., 2023). And the synergistic effects of pyrotinib combined with radiotherapy contribute to the great therapeutic response in our case.

The limitations of our case stem from the absence of tissue or liquid biopsy findings following the resistance of trastuzumab plus pertuzumab, making it challenging to determine the tumor’s genetic status and elucidate the resistance mechanisms. If the patient’s condition deteriorates once more, NGS testing based on tissue or blood specimens is required to identify potential therapeutic targets and exploit alternative salvage therapies. Moreover, after the discovery of scalp and brain metastases in the patient, we implemented a treatment strategy combining mXELIRI chemotherapy with the anti-HER2 drug trastuzumab. However, NGS testing revealed EGFR amplification in the patient. If we had concurrently used an anti-EGFR drug at that time, it might have led to a better therapeutic outcome.

## 4 Conclusion

HER2 is recognized as a promising target for mCRC. In this case report, we presented the case of a patient with treatment-resistant HER2-amplified mCRC who achieved a prolonged response to pyrotinib plus tegafur in combination with radiotherapy following resistance to trastuzumab and pertuzumab. In conclusion, pyrotinib demonstrates activity in the salvage treatment of mCRC patients with HER2 amplification even though resistant to other anti-HER2 drugs. Further studies are needed to validate this finding.

## Data Availability

The original contributions presented in the study are included in the article/supplementary material, further inquiries can be directed to the corresponding author.
